# The Effects of Continuous and Withdrawal Voluntary Wheel Running Exercise on the Expression of Senescence-Related Genes in the Visceral Adipose Tissue of Young Mice

**DOI:** 10.3390/ijms22010264

**Published:** 2020-12-29

**Authors:** Masaki Kimura, Seiya Suzuki, Atsushi Moriya, Kazuki Nogami, Ryoei Uchida, Yoshimasa Saito, Hidetsugu Saito

**Affiliations:** Department of Pharmacotherapeutics, Faculty of Pharmacy, Keio University, 1-5-30 Shibakoen, Minato-ku, Tokyo 105-8512, Japan; seya.szk@gmail.com (S.S.); am.sc846@gmail.com (A.M.); nogamikazuki@gmail.com (K.N.); ryoei920403@gmail.com (R.U.); saito-ys@pha.keio.ac.jp (Y.S.); hidetsugusaito@gmail.com (H.S.)

**Keywords:** legacy effect, exercise, senescence-related gene, SASP, adipose tissue

## Abstract

Obesity has become a global medical problem. The upregulation of senescence-related markers in adipose tissue may cause impairment of adipose tissue and disorders of systemic metabolism. Weight control through diet has been found to ameliorate senescence in the adipose tissue. Exercise is also important in maintaining a healthy lifestyle, however, very few researchers have examined the relationship between senescence-related markers in adipose tissue. Dietary restriction is also reported to have a legacy effect, wherein the effects are maintained for some periods after the termination of the intervention. However, very few researchers have examined the relationship between exercise and senescence-related markers in adipose tissue. Besides, there is no study on the long-term effects of exercise. Hence, we investigated whether the exercise could change the expression of senescence-related genes in the visceral adipose tissue of young mice and whether there was a legacy effect of exercise for 10 weeks after the termination of exercise. Four-week-old male ICR mice were assigned to one of the three groups: 20 weeks of sedentary condition, 20 weeks of voluntary wheel running exercise, or 10 weeks of exercise followed by 10 weeks of sedentary condition. The mice showed decreased expression in genes related to senescence and senescence-associated secretory phenotype, such as *p53*, *p16*, and *IL-6*, in the visceral adipose tissue in response to exercise. These effects were maintained for 10 weeks after the mice stopped exercising. Our study is the first report that exercise reduces the expression of senescence-related genes in the visceral adipose tissue of young mice, and that exercise causes the legacy effect.

## 1. Introduction

Imbalanced energy intake and eating disorders, induced by genetic and environmental factors, can cause overweight and even obesity [1]. Obesity has become a serious global medical problem. Calle et al. [2] reported that obesity increased the risk of death of patients with cardiovascular disease, cancer, or many other diseases. Obesity is also a widely recognized risk factor for premature death; the treatment or prevention of obesity is essential for a long and healthy life.

Recently, Schosserer et al. [3] reviewed age-induced changes in white adipose tissue and concluded that aging caused a time-related decline in cells, organelles, tissues, and the organism that ultimately led to death. Tchkonia et al. [4] reported that the number of senescent cells gradually increased in the adipose tissue of aging mammals. Muñoz-Espín et al. [5] reviewed the accumulation of senescence cells in adipose tissue as one of the major drivers of aging and age-related disorders.

Cellular senescence is the irreversible arrest of cell growth, characterized by the senescence-associated secretory phenotype (SASP) and the staining of senescence-associated β-galactosidase (SA-β-Gal) in cells [4,5]. Senescence is also characterized by increases in senescence-related gene and protein expressions, such as tumor suppressor p53 (encoded by *Trp53* in mice), p21 (also known as WAF1, encoded by *CDKN1A*), and p16 (also known as INK4A, encoded by *CDKN2A*) [5]. Yahagi et al. [6] initially reported that the mRNA and protein levels of p53 were increased in the epididymal fat tissue of young, 12-week-old, genetically obese *ob/ob* mice. Later, several groups reported that senescence-related markers were increased in the white adipose tissue of genetically obese *Ay* mice, high-fat diet-induced obese mice and rats from young, 8 weeks old, to middle age, 10 months old [7,8,9,10,11].

The upregulation of senescence-related markers in adipose tissue also causes the impairment of adipose tissues and disorder of systemic metabolism even in young animals; disrupting the senescence-related markers could reduce the disorder’s severity. For example, Liu et al. [12] showed that the knockout of *MDM2*, the upstream inhibitor of p53, in white adipose tissue led to metabolic disorders, including type 2 diabetes. On the other hand, the metabolic disorders were alleviated in the white adipose tissue of young, *MDM2* and *p53* double knockout mice aged 3–12 weeks. Further, Minamino et al. [7] reported that the inhibition of p53 activity significantly ameliorated insulin resistance in the adipose tissue of the genetically obese, young, 20-week-old, *p53* knockout *Ay* mice. Moreover, Homayounfar et al. [11] reported that the inhibition of p53 using pifithrin, a reversible blocker of p53-dependent transcriptional activation, reverted insulin resistance and normalized insulin sensitivity in high-fat diet-induced young, 17-week-old, obese rats.

On the other hand, weight control appears to ameliorate senescence in adipose tissue. Ishaq et al. [13] reported that dietary restriction ameliorates the level of senescence-related markers, such as p16 and p21, in the visceral adipose tissue of comparatively aged mice (17.5-month-old mice). Similarly, regular exercise, with or without using a free-running wheel, decreases visceral fat volume [14,15,16,17,18]. Exercise is well known to protect against the development of fat cell hypertrophy and insulin resistance [19]. However, very few researchers have examined the relationship between exercise and senescence-related markers in adipose tissue. Schafer et al. [20] firstly reported that exercise prevents diet-induced cellular senescence in adipose tissue using genetically modified middle-aged mice that were almost 1-year-old. They showed a 16-week exercise regimen decreased the expression of *p53*, *p21*, *IL-6*, and *MCP-1* genes in the visceral adipose tissue in the mice on a high-fat, fast-food diet. Meanwhile, exercise had no effect on the normal diet groups.

Therefore, the effects of exercise on the expression of senescence-related genes in the visceral adipose tissue of normal young mice need to be elucidated further. Here, we used microarray analysis to compare the gene expression in normal young mice under sedentary control to those under a voluntary wheel running exercise regimen. Using the gene ontology (GO) analysis and the gene set enrichment analysis (GSEA), we found significant differences in a senescence-related gene set in the visceral adipose tissue between these two conditions (Table 1). 

On the other hand, some dietary interventions reported metabolic memory or legacy effect, wherein the effects were maintained for some periods after the intervention’s termination [21,22,23,24]. Recently, Ishaq et al. [24] reported the metabolic memory of dietary restriction in normal mice adipose tissue. They showed that dietary restriction reduced adipocyte size and sentence-related DNA damage in tissues for at least 3 months after the termination of dietary restriction.

However, there is no study on the long-term effects, or the legacy effects, of exercise on the expression of senescence-related genes in adipose tissue. Therefore, we investigated whether the exercise could change the expression of senescence-related genes in the visceral adipose tissue of normal young mice and whether the effects of exercise could be maintained for 10 weeks after the exercise’s termination.

## 2. Materials and Methods

### 2.1. Animals

Male ICR mice were purchased from Japan SLC Inc. (Shizuoka, Japan). At 4 weeks of age, the mice were divided into three groups according to each group’s comparable mean averaged body weight. The groups were randomly assigned to one of the following 3 groups, 20-week normal control sedentary condition (Co-Co) group (n = 8), 20-week voluntary wheel running exercise (Ex-Ex) group (n = 8), or 10-week exercise followed by 10-week sedentary condition (Ex-Co) group (n = 8). The Ex-Ex and Ex-Co cages included freely accessible running wheels (20 cm in diameter, 65 cm in circumference, and 5 cm in width) with digital revolution counters. All mice groups were fed a regular control diet of 11.5% fat, 60.9% carbohydrate, 27.6% protein per kcal (CE-2, Japan CLEA, Tokyo, Japan), and water ad libitum. All animals were housed individually at 24.5 °C ± 0.1 °C, with 50.3% ± 0.3% humidity and exposed to a 12:12-h light-dark cycle with lights from 8:00 to 20:00. Animal care and experimental procedures were conducted in accordance with the guidelines of Keio University and were approved by the laboratory animal committee (No. 13061, 7 Jul 2019).

### 2.2. Tissue Samples Collection

The mice’s body weights were measured once a week during the 20-week experimental period. At the end of the experimental period, the 24-week-old mice were euthanized by inhaling 1–5% isoflurane. Blood was collected immediately from the abdominal vena cava inferior. Abdominal fat tissue samples, epididymal and retroperitoneal fats, were collected and weighed immediately after euthanasia. We also calculated the sum of these fat-pad weights (= epididymal fat-pad weight (g) × 2 + retroperitoneal fat-pad weight (g) × 2). For mRNA measurement, small pieces of epididymal fat tissue were soaked overnight with the RNA stabilization Solution (RNAlater Solution, Invitrogen, Tokyo, Japan) at 4 °C. After removing the solution, the specimens were stored at –80 °C before analysis.

### 2.3. Total RNA Extraction

Total RNA was isolated from the homogenized mouse epididymal fat tissue with the QIAzol Lysis reagent (QIAGEN, Tokyo, Japan) and purified using the RNeasy Lipid Tissue Mini kit (QIAGEN, Tokyo, Japan) according to the manufacturer’s instructions.

### 2.4. Microarray Analysis

In each group, we chose one mouse whose body weight was closest to the group’s average body weight. Microarray analysis was conducted by Biomatrix Laboratory (Chiba, Japan). The quantity and quality of the extracted total RNAs were assessed by an ND-1000 spectrophotometer (NanoDrop, Tokyo, Japan) and a 2100 Bioanalyzer (Agilent Technologies, Tokyo, Japan), respectively. The total RNAs were then reverse transcribed into fluorescent cRNAs labeled with Cy3 using the Low Input Quick Amp Labeling Kit (Agilent Technologies, Tokyo, Japan). The labeled RNAs were hybridized onto a SurePrint G3 Mouse GE 8×60K Microarray using the Gene Expression Hybridization Kit (Agilent Technologies, Tokyo, Japan). The fluorescent signals were scanned with an Agilent Microarray Scanner and analyzed using the Feature Extraction software (Agilent Technologies, Tokyo, Japan). Each spot’s raw data was normalized by subtracting with the blank spots’ mean background signal. The relative gene expression levels were calculated by comparing the signal intensity of the valid spots from the microarray experiments using the GeneSpring GX v11.5.1 software package (Agilent Technologies, Tokyo, Japan).

### 2.5. Gene Ontology (GO) Analysis and Gene Set Enrichment Analysis (GSEA)

Functional annotation and interpretation were performed to investigate the biological relevance of the significant difference in gene expression between the Co-Co and Ex-Ex groups using the microarray analysis data. We used the Database for Visualization, Annotation, and Integrated Discovery (DAVID, http://david.abcc.ncifcrf.gov/) for comprehensive functional annotation. In this study, we performed GO analysis in the DAVID database to identify statistically significant data (*p* < 0.05) using the genes with a significant change in expression, i.e., more than 2.0- or less than 0.5-fold change compared to the Co-Co condition, from microarray analysis data (Table 1).

Additionally, GSEA was also performed to obtain functional annotation and interpretation using all the gene expression data from the microarray analysis. Based on the size of the set, a normal enrichment score was evaluated for each gene set; FDR and nominal *p* -value were calculated as the cut-off criteria. GSEA was performed using version 7.2 of C2 curated gene sets from online databases. The significantly big changed gene set of “WU_APOPTOSIS_BY_CDKN1A_VIA_TP53” that was significantly related to senescence was presented as a representative data of GSEA (*p* < 0.001).

### 2.6. cDNAs Synthesis and Quantitative Realtime-PCR

Total RNA was reverse transcribed to cDNA using the High-Capacity cDNA Reverse Transcription Kit (Thermo Fisher Scientific, Tokyo, Japan) and the thermal cycler (Nexus Gradient, Eppendorf, Tokyo, Japan). Quantitative real-time PCR was performed using the PowerUp SYBR Green Master Mix (Thermo Fisher Scientific, Tokyo, Japan) in accordance with the manufacturer’s instructions. Quantitative analyses were performed using the CFX96 Real-Time System (CFX Connect, BioRad, Tokyo, Japan). The primer sequences for each of the genes analyzed by quantitative real-time PCR are shown in Supplementary Table S1. The gene 18s ribosomal RNA was used as an internal control.

### 2.7. Statistical Analyses

All data are expressed as mean ± SEM. The differences between groups were analyzed statistically using one-way analysis of variance. Post hoc analysis was performed using Fisher’s Least Significant Difference test when significant main effects were observed. Differences in the running distance between both exercising groups were statistically analyzed using the Student’s t-test. For all tests, *p* < 0.05 was considered statistically significant.

## 3. Results

### 3.1. Animal Conditions

**Figure 1:**
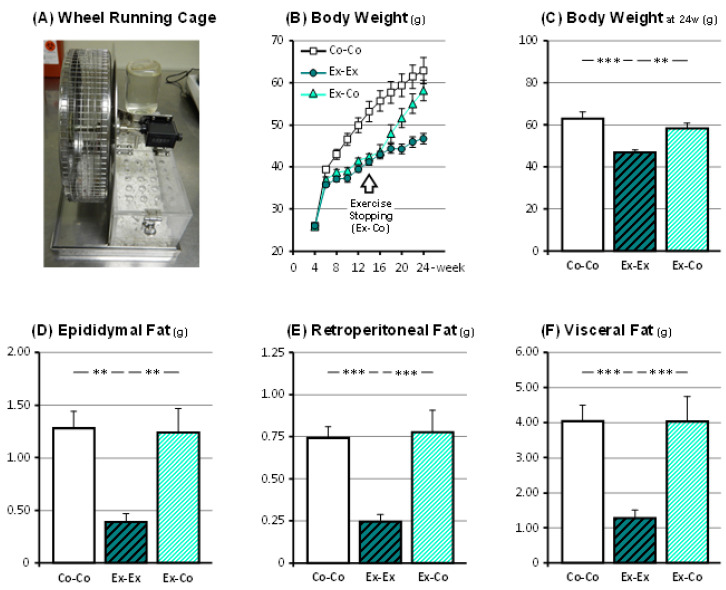
The running wheel cage (**A**), changes in body weight (**B**,**C**), and visceral fat weights, epididymal fat (**D)**, retroperitoneal fat (**E)**, visceral fat (**F**). Co-Co: sedentary control. Ex-Ex: 20-week exercise. Ex-Co: 10-week exercise followed by 10-week sedentary condition groups. Data are represented as mean ± SEM. **: *p* < 0.01, and ***: *p* < 0.001.

The change in the mice’s body weight over time was measured (Figure 1. At week 10, the body weights were significantly lower in the Ex-Ex group, at 41.2 ± 0.7 g (*p* < 0.001), and the Ex-Co group, at 42.5 ± 0.6 g (*p* < 0.001), than that in the Co-Co group, at 53.2 ± 2.5 g. There was no significant difference in body weights between the Ex-Ex and Ex-Co groups. At week 20, the body weights were significantly lower in the Ex-Ex group, at 46.7 ± 1.3 g, than the Co-Co group, at 62.9 ± 3.1 g (*p* < 0.001), and the Ex-Co group, at 58.2 ± 2.4 g (*p* < 0.01). The Ex-Co group’s body weight was reverted to the Co-Co group’s body weight, and no significant difference was found between the groups. Total distance run by the mice was 1,777 ± 142 km, or 12.7 ± 1.0 km/day and 780 ± 55 km, or 11.1 ± 0.8 km/day in the Ex-Ex and Ex-Co group, respectively (*p* < 0.001).

### 3.2. Changes in Fat Tissue Weights

The weights of the epididymal or retroperitoneal fat-pads or their sum were measured (Figure 1). The weight of the Ex-Ex group’s epididymal fat-pads was significantly lower than those of the Co-Co or the Ex-Co groups (*p* < 0.01 for both comparisons); no significant difference was found between the Co-Co and Ex-Co group. The weight of the Ex-Ex group’s retroperitoneal fat-pads was also significantly lower than those of the Co-Co and Ex-Co groups (*p* < 0.001 for both comparisons); no significant difference was observed between the Co-Co and Ex-Co group. We also calculated the sum of these fat-pad weights (= epididymal fat-pad weight (g) × 2 + retroperitoneal fat-pad weight (g) × 2). The sum of these fat-pad weights of the Ex-Ex group was significantly lower than that of the Co-Co and Ex-Co group (*p* < 0.001 for both comparisons), but no significant difference was found between the Co-Co and Ex-Co group.

### 3.3. Changes in mRNA Expression Related to the Adipokine and Glucose Metabolism

**Figure 2:**
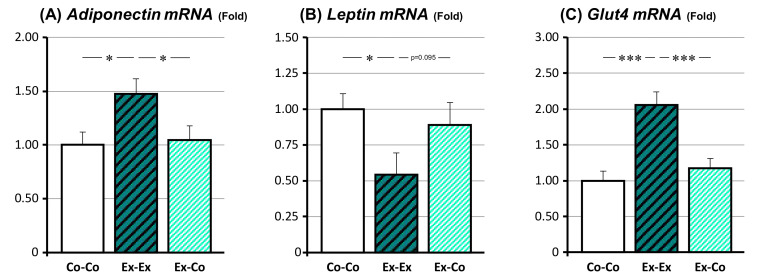
The changes in the expression of adipokine, *adiponectin* (**A**), *leptin* (**B**) and glucose metabolism-related genes, *Glut4* (**C**) in epidydimal fat. Co-Co: sedentary control. Ex-Ex: 20-week exercise. Ex-Co: 10-week exercise followed by 10-week sedentary condition groups. Data are represented as mean ± SEM. Symbols represent *: *p* < 0.05 and ***: *p* < 0.001.

The changes in the mRNA expression of adipokine-related genes, *adiponectin* and *leptin*, and the major glucose transporter in adipose tissue, *Glut4*, were investigated (Figure 2). The Ex-Ex group’s *adiponectin* mRNA expression was significantly higher than that of the Co-Co and Ex-Co groups (*p* < 0.05 for both comparisons), but no significant difference was found between the Co-Co and Ex-Co group. On the other hand, the Ex-Ex group’s *leptin* mRNA expression was lower compared to those of the Co-Co and groups (*p* < 0.05); but no significant difference was found between the Co-Co and Ex-Co group. The *Glut4* mRNA expression of the Ex–Ex group was significantly higher than that of the Co-Co, and Ex-Co groups (*p* < 0.001, respectively), but no significant difference was found between the Co-Co and Ex-Co group.

### 3.4. Changes in mRNA Expression Related to the Senescence

**Figure 3:**
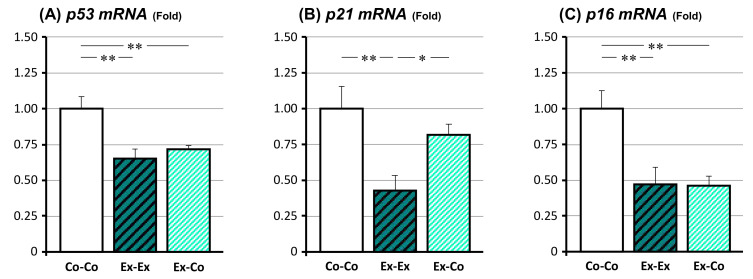
The changes in the expression of senescence-related tumor suppressor genes in epidydimal fat, *p53* (**A**), *p21* (**B**), and *p16* (**C**). Co-Co: sedentary control. Ex-Ex: 20-week exercise. Ex-Co: 10-week exercise followed by 10-week sedentary condition groups. Data are represented as mean ± SEM. Symbols represent *: *p* < 0.05 and **: *p* < 0.01.

The changes in mRNA expression of senescence-related genes, such as *p53*, *p21*, and *p16*, were investigated (Figure 3). The *p53* and *p16* mRNA expressions of the Ex-Ex and Ex-Co groups were significantly lower than those of the Co-Co group (*p* < 0.01, respectively), but there was no significant difference between the Ex-Ex and Ex-Co groups. On the other hand, the Ex-Ex group’s *p21* mRNA expression was significantly lower than that of the Co-Co group (*p* < 0.01). The Ex-Co group’s *p21* mRNA expression showed the tendency to revert to the Co-Co group condition, and therefore, there was a significant difference between the Ex-Ex and Ex-Co groups (*p* < 0.05).

### 3.5. Changes in mRNA Expression Related to the SASP

**Figure 4:**
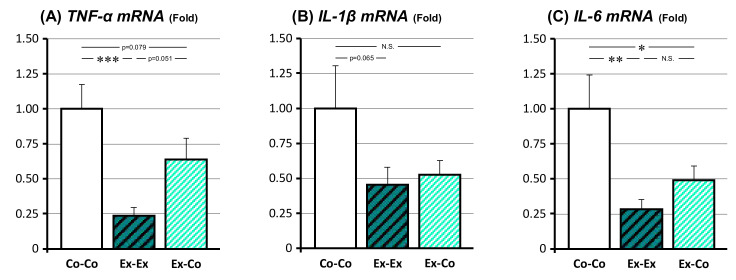
The changes in the expression of senescence-associated secretory phenotype (SASP)-related genes in epidydimal fat, *TNF-α* (**A**), *IL-1β* (**B**), and *IL-6* (**C**). Co-Co: sedentary control. Ex-Ex: 20-week exercise. Ex-Co: 10-week exercise followed by 10-week sedentary condition groups. Data are represented as mean ± SEM. Symbols represent *: *p* < 0.05, **: *p* < 0.01, and ***: *p* < 0.001.

The changes in mRNA expression of the genes associated with SASP, i.e., *TNF-α*, *IL-1β*, and *IL-6*, were examined (Figure 4). The *TNF-α* mRNA expression of the Ex-Ex group was significantly lower than that of the Co-Co group (*p* < 0.001). The *TNF-α* mRNA expression of the Ex-Co group, showed the tendency to revert to the Co-Co group condition (*p* = 0.051 between the Ex-Ex and Ex-Co groups and p = 0.079 between the Co-Co and Ex-Co groups). The *IL-6* mRNA expression of the Ex-Ex and Ex-Co group was significantly lower than that of the Co-Co group (*p* < 0.01 and *p* < 0.05, respectively), but there was no significant difference between the Ex-Ex and Ex-Co group. The *IL-1β* mRNA expressions of the Ex-Ex and Ex-Co groups showed the tendency to decrease compared to the Co-Co group, but no significant difference between the three groups.

## 4. Discussion

This study was designed to clarify the effects of exercise on the expression of senescence-related genes in the visceral adipose tissue. This study also aimed to study the presence of a maintenance period of this effect after exercise was stopped.

This study found a significant decrease in the expression of the senescence and SASP- related genes, i.e., the mRNA level of *p53*, *p16*, and *IL-6* in response to exercise. The effect of exercise also appeared long-lasting, as the reduced level of the genes was maintenance 10-week after exercise was stopped. These mice had reverted to the normal condition as almost the same as the sedentary control mice in body weight, abdominal fat-pad weight, and gene expressions related to adipokine and glucose metabolism. This study is the first to report that exercise ameliorates the expression of senescence-related genes in adipose tissue and that this effect is maintained for a period after stopping exercise.

In this study, a 20-week voluntary wheel running exercise was shown to decrease body weight and visceral adipose tissue significantly compared to the sedentary control; the mRNA expressions of the senescence-related genes were significantly decreased concomitantly. These results are consistent with the findings of Schafer et al. [20], which observed similar effects of exercise on the mRNA level of senescence-related genes *p53*, *p21*, and *IL-6* in visceral adipose tissues using diet-induced obese mice. Recently, many articles have reported that the expression and activation of the senescence-related marker genes were elevated in obese adipose tissue. The p53 and p21 expression in adipose tissue were increased in young, 12-week-old, obese *ob/ob* mice [6]. The gene expression and protein level of p53 and p21 and SA-β-Gal staining were increased in young obese *Ay* mice aged 20 weeks [7]. Similar results using an obese animal model of *ob/ob* or diet-induced obese mice and rats, aged 8 weeks to 10 months, were reported in several articles [8,9,10,11,25]. Even human adipose tissue showed an increased p53 expression in obese subjects [9].

It is well known that obese adipose tissue has inflammation and oxidative stress. In genetically obese mice, B6.Cg *^Ay/+^* and B6.V Lep *^ob/ob^*, and high-fat diet-induced obese mice, M1-polarized proinflammatory macrophages and inflammatory cytokines, such as TNF-α, IL-6, and iNOS, were increased in the perigonadal adipose tissue [26]. The adipose tissue in obese *KKay* mice has severe oxidative stress and reactive oxygen species (ROS), such as an increased level of H_2_O_2_ production, TBARS, and NADPH oxidase subunits, and a decrease in Cu-, Zn-SOD, GPx, and Catalase [27]. Hypoxia was also reported in the adipose tissue of obese animals [28].

Wu et al. [29] reported that TNF-α and NF-κB induced by inflammation increase in p53 activity of Hela cells. Chandel et al. [30] demonstrated that the mitochondrial generation of ROS induced by hypoxia also increases in p53 and p21 levels in human breast carcinoma MCF-7 cells and normal human diploid fibroblast IMR-90 cells. Recently, Vergoni et al. [25] reported that DNA damage, oxidative DNA lesion induced by ROS, contributes to the activation of p53, p21, p16, and p19 in adipose tissue from *ob/ob* and high-fat diet-induced obese mice.

According to previous articles, the *p16* mRNA seems to be one of the most sensitive and reliable senescence-related genes [31,32,33,34,35]. Krishnamurthy et al. [31] reported that, in old, 26-month-old and young, 2.5-month-old mice, the old/young ratio of *p16* mRNA expression was increased substantially in various tissues compared to *p21* mRNA expression. Several articles reported that the elimination of p16-positive cells from several tissues alleviated aging-related diseases and promoted longevity [32,33,34,35]. Recently, Palmer et al. [34] reported that the senolytics, induced by the elimination of p16-positive senescent cells from adipose tissues in genetically modified and pharmacologically treated mice, ameliorated adipose tissue conditions (reduction of cell size, decrease in senescent cells, macrophage infiltration, etc.), and systemic glucose metabolisms (glucose homeostasis and insulin sensitivity under intraperitoneal glucose tolerance test, etc.).

In a study using experimental conditions similar to our study, Schafer et al. [20] reported no significant effects of exercise on the expression of senescence-related genes in adipose tissue. The authors did not observe significant changes in *p53*, *p21*, *p16*, and *IL-6* mRNA expressions between mice under sedentary control conditions and mice under a wireless wheel running exercise regimen. Although they did not show details of this running device, the daily and total running distance of their mice were almost half of the mice in the Ex-Ex group in our study. Furthermore, the mice in the exercise group in their study did not show any significant decrease in the weight of the visceral fat-pads. In our experiment, the mice in the exercise groups showed robust decreases in the weight of their visceral adipose fat-pads compared to the sedentary control mice (Figure 1). As a result, we could demonstrate a significant difference in the expression of senescence-related genes between the mice in the sedentary control and the mice in the exercise groups.

This study showed significant decreases in the expression of senescence and SASP-related genes were maintained after exercising for 10 weeks. However, under the same condition, the bodyweight, abdominal fat-pad weight, and the expressions of genes related to adipokine and glucose metabolism compared to the sedentary control.

The legacy effect induced by exercise was reported previously. Recently, Johnson et al. [36] reported the legacy effects in humans of an exercise intervention on cardiorespiratory fitness and cardiometabolic parameters 10 years after the exercise was stopped. Meanwhile, Haykowsky et al. [37] demonstrated the legacy effects of prior endurance training on the aerobic power and performance in the human heart. However, the legacy effect of exercise on senescence-related gene expressions in adipose tissue was not reported until now. This study is the first to report that exercise ameliorates the expression of senescence-related genes in adipose tissues, and these effects are maintained for a period after exercise is stopped.

On the other hand, the legacy effects of dietary restriction have been reported in many articles. Earlier, Stuchlíková et al. [21] reported long-term effects of a 50% dietary restriction during the first year of life followed by ad-lib feeding increased longevity compared to whole-life ad-lib fed in mice, rats, and golden hamsters. Yu et al. [38] reported that a 40% food restriction in young ages, from age 6 weeks to 6 months, resulted in a small increase, of about 100 days, in longevity in rats. These data suggested the legacy effect of early-life dietary restriction may increase longevity in rodents. Selman et al. [23] reported that an early-life, from age 3 to 11 months, 30% dietary restriction showed metabolic memory in glucose tolerance after reverting to ad-lib feeding for 10 months. Recently, Ishaq et al. [24] reported the metabolic memory of dietary restriction on the expression of senescence-related genes and markers in normal murine visceral adipose tissue. They showed that a 40% dietary restriction reduced adipocyte size and DNA damage related to tissue senescence. The effect was extended for at least 3 months after the dietary restriction ended in comparatively (15-month) old mice.

However, the detailed mechanisms underlying these exercise or dietary restriction-induced legacy effect or metabolic memory have not been studied in these papers. Ceriello [39] discussed some possible mechanisms of metabolic memory in his review. He showed that the suppression of ROS and mitochondrial DNA damage induced by early intensive elimination of advanced glycation end product (AGE) might affect the legacy effect. More recently, Reddy et al. [40] and Kato et al. [41] discussed the epigenetic mechanisms of metabolic memory in their reviews. The legacy effect of exercise interventions may be produced through epigenetic mechanisms related to DNA methylation. Many articles have been reported that exercise alters DNA methylation patterns in many organs, including adipose tissue [42,43,44,45,46]. Voisin et al. [44] reviewed DNA methylations induced by many different types of exercise. Many researchers seem to agree that persistent epigenetic modifications, including DNA methylations triggered by changes in metabolic conditions, can be induced by dietary restriction and exercise, leading to legacy effects and metabolic memory. Rönn et al. [42] reported that 6 months of exercise-induced a significant increase in DNA methylation at the first exon of CpG island of *CDKN2A* (*p16*) gene in adipose tissues. These data suggest, in our study, the continued decrease in the expression of *p16* and other senescence-related genes for a period after the termination of exercise might be induced by DNA methylation induced by exercise in our study.

We think that further investigations are needed to determine the duration of this exercise-induced legacy effect and whether the changes in gene expression could lead to protein synthesis and the body’s systemic function in the near future. Lastly, almost all the parameters, except *p16* mRNA expression, in the Ex-Co group tended to revert to the level in the sedentary control group. Hence, the most important thing about exercise is to continue it for as long as possible.

## 5. Conclusions

We investigated whether the exercise could change the expression of senescence-related genes in the visceral adipose tissue of normal young mice and whether the effects of exercise could be maintained for 10 weeks after the termination of exercise. We found that exercise could lessen the expression of senescence-related genes in murine visceral adipose tissue, and these effects might be maintained for a while after stopping exercise. The mechanisms of exercise-induced legacy effects should be investigated further.

## Figures and Tables

**Table 1:** From gene ontology (GO) analysis, the genes that are significantly up- or down-regulated in the Ex-Ex group compared to Co-Co groups.

**Significantly Up Regulated Gene Sets**	***p*-Value**
Lipid metabolism	0.00023
Glutathione metabolism	0.00720
Gluconeogenesis	0.01700
Adipocytokine signaling pathway	0.02900
Glucose transmembrane transporter activity	0.03700
Glutathione transferase activity	0.03900
Glucose metabolism	0.04500
PPAR signaling pathway	0.04700
**Significantly Down Regulated Gene Sets**	***p*-Value**
Cell cycle	0.00000
Response to cytokine	0.00550
HIF-1 signaling pathway	0.00660
P53 signaling pathway	0.00760
Monocyte chemotaxis	0.01200
Regulation of cytokine secretion	0.01400
Energy reserve metabolic process	0.02000
Macrophage chemotaxis	0.02700

## Supplementary Materials

The following are available online at https://www.mdpi.com/1422-0067/22/1/264/s1.
