# Supplementary material for: The Effects of Continuous and Withdrawal Voluntary Wheel Running Exercise on the Expression of Senescence-Related Genes in the Visceral Adipose Tissue of Young Mice
Source: Int J Mol Sci. 2020 Dec 29;22(1):264. doi: 10.3390/ijms22010264 (PMC7794976; doi:10.3390/ijms22010264)
Supplement: Supplementary file 1 [file ijms-22-00264-s001.pdf]

Supplementary

Table 1. The primers for the genes related to senescence, SASP, adipokine, and glucose metabolism.

Gene name	Forward	Reverse
p53	CCCCAGGATGTTGAGGAGTT	TTGAGAAGGGACAAAAGATGACA
p21	ACTTCCTCTGCCCTGCTGC	GGTCTGCCTCCGTTTTCG
p16	ATGGAGTCCGCTGCAGACAG	ATCGGGGTACGACCGAAAG
TNF-a	GTTCTATGGCCCAGACCCTCAC	GGCACCACTAGTTGGTTGTCTTTG
IL-1b	TGTTTTCCTCCTTGCCTCTGAT	GAGTGCTGCCTAATGTCCCCTT
IL-6	CCACTTCACAAGTCGGAGGCTTA	GCAAGTGCATCATCGTTGTTCATAC
Adiponectin	GTCAGTGGATCTGACGACACAA	ATGCCTGCCATCCAACCTG
Leptin	ACCTGTCTACTCATGCCGCACTC	CTGTCCTGCAGCCTGTTTG
Glut4	CAACAGCTCTCAGGCATCAA	ACCGAGACCAACGTGAAGAC
18s rRNA	TTGACGGAAGGGCACCACCAG	GCACCACCACCCACGGAATCG
